# Supplementary material for: A Lesson from the Green Pass Experience in Italy: A Narrative Review
Source: Vaccines (Basel). 2022 Sep 6;10(9):1483. doi: 10.3390/vaccines10091483 (PMC9505186; doi:10.3390/vaccines10091483)
Supplement: Supplementary file 1 [file vaccines-10-01483-s001.zip › vaccines-1870859-supplementary.pdf]

Table S1. Mach search terms.

Mach Search Terms	No. records PubMed	No. records Scopus	No. records Web of Science
**(covid AND vaccine AND "green pass" AND Italy)**	8	7	8
**(covid AND vaccine AND certificate AND Italy)**	18	6	12
**(covid AND vaccine AND passport AND Italy)**	8	2	4
**Total**	**73**		
